# No change in network connectivity measurements between separate rsfMRI acquisition times

**DOI:** 10.3389/fnetp.2024.1342161

**Published:** 2024-01-15

**Authors:** Elliot G. Neal, Samantha Schimmel, Zeegan George, Molly Monsour, Adam Alayli, Gavin Lockard, Keaton Piper, Stephanie Maciver, Fernando L. Vale, Yarema B. Bezchlibnyk

**Affiliations:** ^1^ Department of Neurosurgery and Brain Repair, University of South Florida Morsani College of Medicine, Tampa, FL, United States; ^2^ Department of Neurology, Advent Health Tampa, Tampa, FL, United States; ^3^ Department of Neurosurgery, Medical College of Georgia, Augusta University, Augusta, GA, United States

**Keywords:** resting state, functional MRI, epilepsy, neurosurgery, network

## Abstract

The role of resting state functional MRI (rsfMRI) is increasing in the field of epilepsy surgery because it is possible to interpolate network connectivity patterns across the brain with a high degree of spatial resolution. Prior studies have shown that by rsfMRI with scalp electroencephalography (EEG), an epileptogenic network can be modeled and visualized with characteristic patterns of connectivity that are relevant to both seizure-related and neuropsychological outcomes after surgery. The aim of this study is to show that a 5-min acquisition time provides reproducible results related to the relevant connectivity metrics when compared to a separately acquired 5-min scan. Fourteen separate rsfMRI sessions from ten different patients were used for comparison, comprised of patients with temporal lobe epilepsy both pre- and post-operation. Results showed that there was no significant difference in any of the connectivity metrics when comparing both 5-min scans to each other. These data support the continued use of a 5-min scan for epileptogenic network modeling in future studies because the inter-scan variability is sufficiently low as not to alter the output metrics characterizing the network connectivity.

## 1 Introduction

### 1.1 Resting state fMRI in epilepsy surgery

Epilepsy remains a major public health burden, with an estimated global prevalence of 0.5%–1% and a national prevalence of 1.2% (Tellez-Zenteno and Hernandez-Ronquillo, 2012; Zack and Kobau, 2017). Unfortunately, up to 30% of patients experience medically refractory epilepsy and continue to have seizures despite adequate trials of 2 or more anti-seizure medications (Strzelczyk et al., 2017; Neal et al., 2019). These patients may subsequently seek resective epilepsy surgery, which disconnects epileptogenic tissue and is curative in 70%–80% of patients (Bell et al., 2009; Chang et al., 2015; Neal et al., 2020). Seizure freedom is heavily influenced by rigorous localization of a discrete epileptogenic zone and its relationship to eloquent areas (Bell et al., 2009; Morgan et al., 2010). In fact, 80% of patients with visualizable lesions on structural magnetic resonance imaging (MRI) experienced seizure freedom, versus 18%–63% of patients without evident structural abnormalities (Bell et al., 2009; Morgan et al., 2012). While structural MRI and electroencephalogram (EEG) remain the current gold standard for pre-operative epilepsy surgery planning, EEG is limited by the spatial resolution and depth of penetration, while MRI can only detect radiographically apparent structural abnormalities (Thornton et al., 2010; Pizarro et al., 2016; Chen et al., 2017).

Resting state functional MRI (rsfMRI) detects brain functional connectivity based on the temporal correlation of blood oxygenation level dependent (BOLD) signal changes at rest and has emerged as a non-invasive technique for pre-operative epileptogenic zone localization (Bettus et al., 2010; Morgan et al., 2012). By identifying regions with concurrent BOLD fluctuations, rsfMRI enables the identification of functional networks (Bettus et al., 2010). Given the paradigm shift recognizing epilepsy as a disorder of network dysfunction, localizing connectivity of epileptogenic networks may aid neurosurgeons in the resection of epileptogenic tissue necessary and may be sufficient for the achievement of seizure freedom (Pizarro et al., 2016). Recent studies have addressed the utility of rsfMRI in preoperative planning, with Chen et al. describing rsfMRI to be as specific as EEG in localizing epileptogenic tissues and Bettus et al. reporting the ability of rsfMRI to detect altered functional connectivity in both the epileptic and non-epileptic hemispheres of patients with mesial temporal lobe epilepsy (Bettus et al., 2010; Chen et al., 2017). Additionally, rsfMRI aids in determining the extent of seizure network involvement and may predict postoperative seizure outcomes (Neal et al., 2019). Thus, rsfMRI remains a promising tool in the clinical management of patients with medically refractory epilepsy.

### 1.2 Duration of rsfMRI acquisition

In addition to conventional neuroimaging challenges such as cost, availability, and patient cooperation, rsfMRI acquisition presents unique challenges because individuals must stay awake while avoiding focusing on any mental activities (Anderson et al., 2011; Elliott et al., 2019). Typically, a rsfMRI acquisition requires a 3.0 Tesla scanner and concurrent volumetric structural MR imaging for co-registration with brain models (Neal et al., 2018). One parameter important for obtaining ample rsfMRI data is the duration of the rsfMRI acquisition. Thus far, there is no clear consensus regarding the optimal acquisition time. Some studies describe adequate measurements of functional connectivity with 5–7-min scans (Van Dijk et al., 2010; Termenon et al., 2016; Moreno-Ayure et al., 2020; Raimondo et al., 2021) while others report increased reliability of scans from 5 to 13 min (Anderson et al., 2011; Birn et al., 2013; Elliott et al., 2019). Additionally, Van Djik et al., found that multiple short scans provided the same results as a long continuous scan, a finding beneficial in the context of patient comfort and scanner availability (Van Dijk et al., 2010). As previously published, we typically conduct rsfMRI with a BOLD MRI sequence for runs of 5 min with a repetition time (TR) of 3,000 ms and an echo time (TE) of 35 ms (Neal et al., 2018).

### 1.3 Objective

In the present study, we aim to validate our center’s 5-min rsfMRI acquisition protocol by comparing network connectivity data generated using two 5-min rsfMRI scans acquired at different times. We hypothesize that our previously published analyses, including global brain connectivity, intra-hemispheric connectivity, and connectivity and spread of positively and negatively correlated epilepsy networks will not differ between the separately acquired 5-min rsfMRI scans. This would support the hypothesis that the signal to noise ratio is adequately high to generate reproducible data points from a single 5-min scan.

## 2 Methods

### 2.1 Patient demographics

All reported data followed the Strengthening the Reporting of Observational studies in Epidemiology (STROBE) guidelines for observational trials and the protocol and informed consent was approved by our university’s Institutional Review Board (IRB). Fourteen separate sessions, each consisting of two 5-min rsfMRI acquisitions were included for analysis. All patients were diagnosed with temporal lobe epilepsy and underwent surgery consisting of either open microsurgery, laser interstitial thermal therapy for amygdalohippocampotomy, or neuromodulation. Eleven rsfMRI sessions were from patients before surgery, and three were from after the surgery. The patients included in this study signed consent and agreed to participate in this study between June 2014 to December 2022, and the date of surgery may fall outside of this range. In addition to the rsfMRI studies detailed above, each patient underwent a standard pre-surgical evaluation for epilepsy surgery including MRI, epilepsy monitoring unit (EMU) video-EEG, Wada testing, (18F-FDG) PET, and neuropsychological testing.

### 2.2 Data acquisition

Two 5-min rsfMRI acquisitions with the same parameters were obtained, one at the beginning and the other at the end of the MRI session. rsfMRI was conducted in a 3-Tesla MRI with a blood oxygenation level dependent (BOLD) MRI sequence. rsfMRI was acquired with the patient lying supine with eyes closed. Each rsfMRI sequence consisted of a single 5-min acquisition with parameters as follows: echo time (TE) of 35 ms, repetition time (TR) of 3000 ms, and a voxel size of 4 × 3.75 × 3.75 mm. Volumetric non-contrasted T1-weighted thin slice MRI was acquired during the same session. The first 5-min scan was used in the network modeling algorithm and compared to data generated from the second 5-min acquisition.

### 2.3 Network modeling

**FIGURE 1:**
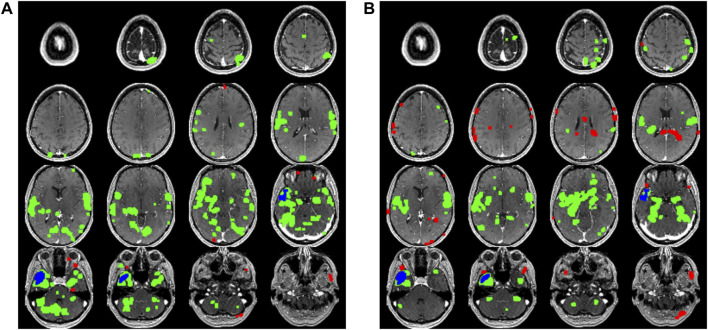
A comparison series of axial slices through a T1 weighted MRI scan of one of the patients included in the study is shown. The blue voxels represent the putative epileptogenic zone generated from the surface EEG inverse source localization. The green voxels correspond to the positively correlated epilepsy network while the red voxels correspond to the negatively correlated epilepsy network. **(A)** Data generated from the first 5 min rsfMRI study are shown compared to **(B)** the second 5 min study.

The epilepsy network for each patient was modeled as previously described to create an anatomical localization of the epileptogenic zone (Figure 1) (Neal et al., 2018). Briefly, all MR image sets were motion corrected, smoothed, and transformed into Montreal Neurological Institute (MNI) space using the six-parameter rigid body spatial transformation algorithm and co-registered using SPM12 (Wellcome Department of Imaging Neuroscience, University College London, United Kingdom). The scalp EEG data were filtered to remove non-physiologic frequencies and cropped to include only the inter-ictal or ictal signals identified by a blinded neurophysiologist (MATLAB 2023; Natick, MA). Ictal and inter-ictal source discharges were localized by first generating a transformed mesh representing the patient-specific anatomy from which a model could be formed from the thin-slice T1-weighted MRI sequence. Then, cortical dipoles were modeled using a forward computation, followed by an empirical Bayesian approach to inverse reconstruction, localizing the theoretical evoked response (SPM12). This process was used to generate a hypothesized epileptogenic or irritative zone source volume, which was co-registered to the rsfMRI in MNI space. A connectivity matrix of Pearson correlation values for each voxel in the brain was computed and averaged for the entire brain as well as for the hemisphere ipsilateral or contralateral to the hemisphere in which surgery was done, and this data was recorded as the “global connectivity,” “ipsilateral hemisphere connectivity,” and “contralateral hemisphere connectivity” for purposes of data analysis.

The rsfMRI time-series signature was extracted from the epileptogenic zone volume and individual intra-axial image voxels to generate a Pearson correlation coefficient for each voxel with respect to the epileptogenic zone. At this point, each voxel in the brain has an associated Pearson correlation value ranging from −1 to 1, which can be thought to roughly represent how similarly each voxel behaves when compared to the epileptogenic zone, therefore representing an estimation of similarity in firing patterns and perhaps in connectedness within a similar network. When an appropriate threshold as described in previous publications is applied, a positively and negatively correlated epilepsy network is generated consisting of a collection of voxels presumed to be included in a network correlated or anticorrelated with the epileptogenic zone (Neal et al., 2020; Neal et al., 2021). The number of voxels included in the network is recorded as the “size” of the network. Next, the Euclidean distance between each of these voxels and the centroid of the epileptogenic zone as measured in voxel length is calculated to estimate the “spread” of the network. Each voxel within the network is compared to every other voxel in the network and a connectivity matrix of Pearson correlation values is calculated to estimate the within-network connectivity of both the positively and negatively correlated epilepsy network, which is referred to as the “network connectivity”.

### 2.4 Statistical analysis

A two-sample paired *t*-test was used to compare independent groups with continuous variables. *p*-values less than alpha = 0.05 were considered significant. Bonferroni correction was used to correct for multiple comparisons, resulting in alpha = 0.0056 (9 independent comparisons performed consisting of the 9 output variables listed in Table 1. Network modeling statistical tests were conducted using IBM SPSS Statistics Version 26 (IBM Corp., Armonk, New York, United States).

**TABLE 1:** Network connectivity comparison.

	Five minute rsfMRI 1		Five minute rsfMRI 2		
	Mean	Std. Deviation	Mean	Std. Deviation	*p*-value
Global Connectivity (Pearson Correlation)	0.3780	0.1177	0.3130	0.1291	0.1751
Ipsilateral Hemisphere Connectivity (Pearson Correlation)	0.3757	0.1113	0.3236	0.1452	0.3561
Contralateral Hemisphere Connectivity (Pearson Correlation)	0.3738	0.1157	0.3205	0.1328	0.3275
Positively Correlated Network Size (Voxels)	6839	1384	7615	1526	0.4239
Negatively Correlated Network Size (Voxels)	719.8	1165	2881	5517	0.4163
Positively Correlated Network Connectivity	0.5068	0.2566	0.4732	0.2783	0.8323
Negatively Correlated Network Connectivity	0.3019	0.0884	0.2835	0.1737	0.8218
Positively Correlated Network Spread (Median)	36.95	4.643	38.35	5.453	0.6416
Negatively Correlated Network Connectivity	43.06	7.049	42.62	7.739	0.9187

## 3 Results

### 3.1 Demographics

A total of 14 separate MRI sessions from 10 unique patients were used for analysis, consisting of 10 sessions from the pre-operative workup, 3 from the post-operative 3-month follow up, and 1 from the 1-year post-operative follow up visits. The post operative sessions used were all from patients from whom pre-operative sessions were also included. Seven (50%) of the sessions were from male patients and remainder were from females. All ten patients were diagnosed with temporal lobe epilepsy by an interdisciplinary epilepsy surgery team. Eight patients (80%; 8 fMRI sessions, 57%) had mesial temporal sclerosis read by a radiologist on the preoperative MRI. Six patients (60%; six fMRI sessions, 43%) had surgery on the left hemisphere while the remainder had surgery on the right side. Surgeries consisted of laser interstitial thermal therapy for amygdalohippocampotomy (5 patients, 50%; 8 fMRI sessions, 57%) hippocampal sparing amygdalohippocampectomy (2 patients, 20%; 2 fMRI sessions, 14%), thalamic anterior nucleus deep brain stimulation (1 patient, 10%; 1 fMRI session, 7%), and anterior temporal lobectomy (2 patients, 20%; 2 fMRI sessions, 14%).

### 3.2 No difference in global network measurements

First, the network connectivity measured using Pearson correlation coefficients across the entire brain was measured and compared between the two 5-min rsfMRI acquisitions; these data are shown in Table 1. There was no significant difference between the two 5-min scans (mean 0.3780 ±0.12 vs. 0.3130±0.13, *p* = 0.1751). Average connectivity in the entire hemisphere ipsilateral to the surgery performed was also compared, and no significant difference was found between the 2 5-min acquisitions (mean 0.3757±0.11 vs. 0.3236±0.15, *p* = 0.3561). Connectivity in the hemisphere contralateral to the surgery was also not significantly different between the 2 5-min acquisitions (mean 0.3738±0.12 vs. 0.3205±0.13, *p* = 0.3275).

### 3.3 No difference in epilepsy network specific measurements

Next, the positively and negatively correlated epilepsy network was modeled using both separately acquired rsfMRI acquisitions. The number of voxels in the positively correlated network (mean 6839±1384 vs. 7615±1526, *p* = 0.4239) and the negatively correlated network (mean 720±116 vs. 2881±5517, *p* = 0.4163) were not significantly different between both 5-min scans. Average connectivity within both the positively correlated (mean 0.5068±0.26 vs. 0.4732±0.28, *p* = 0.8323) and negatively correlated epilepsy network (mean 0.3019±0.09 vs. 0.2835±0.17, *p* = 0.8218) were not significantly different between two 5-min acquisitions. Spread of the network across the brain was measured by the median distance between the voxels in the epilepsy network and the center of the irritative zone, and was not found to be significantly different between the 2 5 minute rsfMRI scans in either the positively correlated network (mean 36.9±4.64 vs. 38.35±5.45, *p* = 0.6416) or the negatively correlated network (mean 43.1±7.05 vs. 42.6±7.74, *p* = 0.9187).

## 4 Discussion

In this brief report, we demonstrate that a 5-min rsfMRI acquisition generates stable epilepsy network connectivity data regardless of when it is acquired during an MR imaging session. We compared several previously reported measures, including global brain connectivity, connectivity within each hemisphere, and connectivity and spread of a previously described positively and negatively correlated epilepsy network. All other variables were controlled, and the patients were comprised of both preoperative and postoperative cases with temporal lobe epilepsy. If the output metrics were outpaced by the noise and variability between two separate scans, then it would be necessary to increase the scan time to increase the signal to noise ratio.

The aim of the present study was to validate our established method for non-invasive network modeling with the independent variable as the length of the MRI scan. In future studies, we aim to control other variables including image smoothing and motion correction, atlas registration parameters, EEG source localization algorithm, and in the post-processing of MRI and EEG data in order to support a robust and reproducible network model that hopefully will be useful in planning epilepsy surgeries. We then hope to expand our use of the modeling algorithm to continue investigations in other various aspects of surgical outcomes related to network characteristics.

The main limitation of this study is that it cannot be said that the 5-min rsfMRI study is universally adequate for any potential network modeling scenario. We only tested for the specific output that we have studied in past publications and were not able to test all possible permutations of the network modeling algorithm or output measurements. If it is decided that new parameters characterizing the network modeling results will be studied, then the shorter 5-min rsfMRI will have to be validated for that particular use-case. As such, a limitation of the study is that more output metrics were not used which limits the external validity of this study on other rsfMRI network modeling studies.

This brief report serves as a benchmark to support the use of a 5-min rsfMRI sequence in epilepsy network modeling using non-invasive methodology previously described (Neal et al., 2018; Neal et al., 2019; Neal et al., 2020; Neal et al., 2021).

## Data Availability

The raw data supporting the conclusion of this article will be made available by the authors, without undue reservation.
